# Curcumin‐activated Wnt5a pathway mediates Ca^2+^ channel opening to affect myoblast differentiation and skeletal muscle regeneration

**DOI:** 10.1002/jcsm.13535

**Published:** 2024-07-10

**Authors:** Mao‐yuan Wang, Jia‐ming Yang, Yi Wu, Hai Li, Yan‐biao Zhong, Yun Luo, Rui‐lian Xie

**Affiliations:** ^1^ Department of Rehabilitation Medicine First Affiliated Hospital of Gannan Medical University Ganzhou China; ^2^ Ganzhou Key Laboratory of Rehabilitation Medicine Ganzhou China; ^3^ Key Laboratory of Prevention and Treatment of Cardiovascular and Cerebrovascular Diseases, Ministry of Education Gannan Medical University Ganzhou China; ^4^ Ganzhou Intelligent Rehabilitation Technology Innovation Center Ganzhou China; ^5^ Department of Oncology First Affiliated Hospital of Gannan Medical University Ganzhou China

**Keywords:** Ca^2+^, Curcumin, Muscle regeneration, Myoblast differentiation, Wnt5a

## Abstract

**Background:**

Skeletal muscle injury is one of the most common sports injuries; if not properly treated or not effective rehabilitation treatment after injury, it can be transformed into chronic cumulative injury. Curcumin, an herbal ingredient, has been found to promote skeletal muscle injury repair and regeneration. The Wnt5a pathway is related to the expression of myogenic regulatory factors, and Ca^2+^ promotes the differentiation and fusion process of myoblasts. This study explored the effect and mechanism of curcumin on myoblast differentiation during the repair and regeneration of injured skeletal muscle and its relationship with the Wnt5a pathway and Ca^2+^ channel.

**Methods:**

Myogenic differentiation of C2C12 cells was induced with 2% horse serum, and a mouse (male, 10 weeks old) model of acute skeletal muscle injury was established using cardiotoxin (20 μL). In addition, we constructed a Wnt5a knockdown C2C12 cell model and a Wnt5a knockout mouse model. Besides, curcumin was added to the cell culture solution (80 mg/L) and fed to the mice (50 mg/kg). Fluorescence microscopy was used to determine the concentration of Ca^2+^. Western blot and RT‐qPCR were used to detect the protein and mRNA levels of Wnt5a, CaN, NFAT2, MyoD, Myf5, Pax7, and Myogenin. The expression levels of MyoD, Myf5, Myogenin, MHC, Desmin, and NFAT2 were detected using immunofluorescence techniques. In addition, MyoD expression was observed using immunohistochemistry, and morphological changes in mouse muscle tissue were observed using HE staining.

**Results:**

During myoblast differentiation and muscle regeneration, Wnt5a expression was upregulated (*P* < 0.001) and the Wnt5a signalling pathway was activated. Wnt5a overexpression promoted the expression of MyoD, Myf5, Myogenin, MHC, and Desmin (*P* < 0.05), and conversely, knockdown of Wnt5a inhibited their expression (*P* < 0.001). The Wnt5a pathway mediated the opening of Ca^2+^ channels, regulated the expression levels of CaN, NFAT2, MyoD, Myf5, Myogenin, MHC, and Desmin (*P* < 0.01) and promoted the differentiation of C2C12 myoblasts and the repair and regeneration of injured skeletal muscle. The expression of Wnt5a, CaN, NFAT2, MyoD, Myogenin, Myf5, and MHC in C2C12 myoblast was significantly increased after curcumin intervention (*P* < 0.05); however, their expression decreased significantly after knocking down Wnt5a on the basis of curcumin intervention (*P* < 0.05). Similarly, in Wnt5a knockout mice, the promotion of muscle regeneration by curcumin was significantly attenuated.

**Conclusions:**

Curcumin can activate the Wnt5a signalling pathway and mediate the opening of Ca^2+^ channels to accelerate the myogenic differentiation of C2C12 cells and the repair and regeneration of injured skeletal muscle.

## Introduction

Skeletal muscle injury is one of the most common sports injuries, and it often affects an athlete's physical function and athletic performance. Finding ways to promote rapid recovery from skeletal muscle injury will help improve their athletic performance. Skeletal muscle is a highly plastic tissue with a strong capacity to regenerate after injury. It is important to note that regeneration after skeletal muscle injury is a complex process involving various cell populations, the precise regulation of gene expression and growth factors and connective tissue components.^1^ Therefore, the exploration of the molecular mechanisms regulating skeletal muscle regeneration and the methods to effectively promote skeletal muscle regeneration will provide strategies for accelerating recovery after sports injuries.

The regenerative capacity of skeletal muscle depends on skeletal muscle stem cells—satellite cells—that are located between the myocyte membrane and basement membrane of skeletal muscle and are considered to be the predominant myogenic stem cells in muscle repair.^2^ When skeletal muscle is injured, quiescent satellite cells undergo activation, proliferation, differentiation, and fuse with damaged muscle fibres to repair it under the regulation of cytokines and growth factors.^3^ Myogenic regulatory factors (MRFs) are the primary regulators of satellite cell proliferation and differentiation. MRFs mainly include four members: MyoD, Myf5, MRF4, and Myogenin, which are required for the differentiation of myoblasts.^4^ In addition, the myosin heavy chain (MHC) is considered a marker of myotube formation,^5^ and Desmin is the major intermediate filament of skeletal muscle, contributing to muscle structure and cell integrity, force transmission, and mitochondrial homeostasis.^6^ Therefore, exploring the factors affecting the expression of these molecules is of great significance to elucidate the specific mechanism of myoblast differentiation and the treatment of skeletal muscle damage.

Numerous studies have shown that the Wnt signalling pathway plays a key regulatory role in the processes of cell proliferation, differentiation, and migration.^7^, ^8^ Moreover, the Wnt signalling pathway has been shown to be involved in the development and regeneration of skeletal muscle.^9^ Wnt5a, an important member of the Wnt family, has been shown to be involved in the myogenic differentiation process of C2C12 cells.^10^ We have already mentioned that MRFs are essential for myoblast differentiation, so can Wnt5a mediate the myogenic differentiation process of C2C12 cells by regulating the expression of MRFs? It has been shown that in myoblasts, elevated intracellular Ca^2+^ concentration is essential for the differentiation of myoblasts and fusion of myotubes.^11^ Furthermore, Wnt5a can mediate neuronal development and differentiation by regulating the Ca^2+^/calpain signalling pathway.^12^ Further studies have shown that the Wnt5a/Ca^2+^/CaN/NFAT signalling pathway plays a key role in embryonic finger development and is a permissive factor for cartilage formation in vivo.^13^ Among them, calcineurin (CaN) can be activated by Ca^2+^, and the activated CaN can induce dephosphorylation of nuclear factor of activated T cells (NFAT) and translocation to the nucleus to exert physiological effects.^14^ Therefore, does the Wnt5a/Ca^2+^/CaN/NFAT signalling pathway play an equally important role in the process of myogenic differentiation and muscle regeneration? This is the first important question to be explored in this study.

Curcumin is a polyphenolic compound contained in turmeric, which has been reported to show significant effects on the differentiation of myoblasts, and it promotes the differentiation of mouse primary myoblasts in vitro.^15^ In addition, curcumin has a significant protective effect on skeletal muscle injury.^16^ However, the specific mechanism of action of curcumin in myoblast differentiation and skeletal muscle regeneration is still unclear. Numerous studies have shown that curcumin is closely linked to the Wnt5a signalling pathway.^17^, ^18^ These findings suggest that curcumin can regulate Wnt5a and thus mediate disease progression and that Wnt5a has been shown to be involved in myogenic differentiation of C2C12 cells,^19^ as well as the Wnt5a/Ca^2+^ signalling pathway has been found to be closely related to myogenic differentiation.^20^ Moreover, the Wnt5a/Ca^2+^/CaN/NFAT signalling pathway, which we mentioned earlier, plays an important role in the process of cartilage differentiation.^13^ Therefore, we hypothesized that curcumin promotes the expression of Wnt5a and activate the Wnt5a/Ca^2+^/CaN/NFAT signalling pathway, thus promoting the process of myogenic differentiation and muscle regeneration. This is the second important question that this study seeks to explore.

In conclusion, this study aims to clarify the mechanism of the influence of curcumin on the differentiation of myoblasts by activating the Wnt5a pathway and mediating the opening of Ca^2+^ channel, which will provide good therapeutic strategies and ideas for the regeneration and repair of skeletal muscle after injury.

## Materials and methods

### Cell culture

C2C12 myoblasts were purchased from Shanghai Cell Bank, Chinese Academy of Sciences. The growth medium was DMEM containing fetal bovine serum (10%) and penicillin/streptomycin (1%). Differentiation medium was prepared by adding 2% horse serum to the growth medium. The cell culture incubator parameters were 37°C and 5% CO_2_. Cell morphology was observed by microscopy, and cell passages were performed by routine change of culture medium.

### Establishment of the C2C12 myoblast differentiation model

Purchased C2C12 myoblasts were cultured in six‐well plates at a cell density of 1 × 10^5^ per dish and cultured in growth medium. At approximately 80% cell fusion, differentiation medium was substituted for growth medium to induce myogenic differentiation. The medium was changed every 2 days. The C2C12 myoblast differentiation model was obtained by continuous induction and differentiation for 6 days and was used for biochemical analysis. In addition, C2C12 myoblasts were transfected with Wnt5a overexpression and knockdown lentiviral vectors and controls, and the changes in various indicators during the induction of differentiation were detected. Moreover, 80 mg/L of curcumin solution was added to the differentiation medium for 24 h to observe the effect of curcumin on the myogenic differentiation of C2C12 cells.

### Establishment of a mouse model of skeletal muscle injury and repair

Ten‐week‐old 23–25 g male C57BL/6 mice were purchased from the Animal Experimental Center of Kunming Medical University (License No.: SCXK (Dian) K2020‐0004). Wnt5a knockout mice (C57BL/6) were obtained from Southern Model Biotechnology Co., Ltd. (Shanghai, China). The skeletal muscle injury and repair model were induced by cardiotoxin (CTX); 1 mg CTX dry powder was dissolved in 2224 μL sterile 0.9% NaCl solution and configured as 500 μM CTX solution. During modelling, sterile 0.9% NaCl was used to diluted it to 10 μM (1:50 dilution), and each mouse was injected with 20 μL 10 μM CTX. Mice were anaesthetized with 3% pentobarbital sodium (40 mg/kg), their hind limbs were shaved and washed with alcohol, and CTX was injected into the anterior tibial muscle with a 26‐gauge needle. The mice were killed randomly at each time point on the 1st, 3rd, 5th, 7th, 9th, 11th, 14th, and 21st days after CTX injection (*n* = 5 for each group), and skeletal muscle injury, regeneration, and repair were observed. In addition, 2 days after CTX injection, mice in the curcumin group were administered 50 mg/kg curcumin (concentration 12.5 mg/mL) once daily by gavage, while the control group was given equal amounts of corn oil, and mice were sacrificed on days 1, 7, and 14 after gavage, respectively. The collected muscle tissues were divided into three parts and then used for western blot, RT‐qPCR, and histological examination respectively. All animal experiments in this study were approved by the Biomedical Research Ethics Committee of Gannan Medical University (ethics number: 2020363).

### Western blot

The tissue was first frozen and ground in liquid nitrogen and then lysed in RIPA lysis solution (Beyotime, China). The protein concentration was detected by a BCA reaction kit. After quantitative analysis, the total protein was boiled at 100°C to denature the protein. The SDS–PAGE method was used for protein electrophoresis, the electrophoresis apparatus (Bio‐RAD, USA) was adjusted from 90 to 120 V, a PVDF membrane (Millipore, USA) was used for membrane transfer, and skim milk (Sigma, USA) was used for blocking. Pre‐diluted primary antibodies against Wnt5a, CaN, NFAT2, MyoD, Myf5, myogenin, Pax7, and β‐actin (1:1000; Abcam, UK) were incubated for 10 h at 4°C with slow shaking. The next day, goat anti‐mouse antibody or goat anti‐rabbit antibody (1:2000; Abcam, UK) was incubated for 1 h with slow shaking at indoor temperature. ECL chemiluminescence solution was used for development, a chemiluminescence instrument was used for exposure and observation, and ImageJ was used for protein band analysis.

### RT‐qPCR

A total RNA kit was used to obtain total RNA, which was treated with DNase I (Takara, Japan). Care was taken to avoid RNA degradation and contamination during extraction. The tissues were first frozen and ground in liquid nitrogen, and then TRIzol reagent was added to isolate total RNA. The ratio of OD260/OD280 was used to identify the total RNA purity by Nano Drop. Fluorescence quantitative PCR was performed using cDNA with the following conditions: pre‐denaturation at 95°C for 20 s, and then the amplification cycle was carried out at 95°C for 1 s and 60°C for 20 s, and there were 40 cycles in this stage. Then, a dissolution curve analysis stage was performed, wherein the temperature of the dissolution curve was set to 60–95°C, and each sample was provided with three duplicate wells. Using β‐actin as a reference control, the level of the target product relative to the internal control was expressed as 2^−ΔΔCt^. The primer sequences for the genes are shown in Table S1.

### Immunofluorescence

Using PBS to wash the slides to be tested and then using 4% paraformaldehyde to fixed it for 15 min. Diluted primary antibodies MyoD, Myf5, Myogenin, MHC, Desmin, and NFAT2 (1:100; Abcam, UK) and reacted at 37°C for 0.5 h. After that, using PBS to wash it three times, and dripped with goat anti‐mouse secondary antibody (1:1000; Abcam, USA) labelled with fluorescent, incubated at 37°C for 0.5 h. Using DAPI to stain the nuclei, and incubating for 5 min in the dark. A fluorescence microscope was used to observe and collect the images.

### HE staining

The collected muscle tissue was fixed with 4% paraformaldehyde. The tissues were then dehydrated step by step with ethanol solutions of different concentrations (70%–80%–90%–95%–100%–100%). The tissues were then sequentially soaked in xylene for transparency. The tissue is then soaked in paraffin. The wax‐impregnated tissue blocks were sliced after the embedding treatment. The sections were dewaxed and hydrated with xylene and gradient ethanol, haematoxylin staining, hydrochloric acid differentiation, running water washing, eosin staining, running water washing, gradient ethanol and xylene dehydration and transparency, drying at room temperature, dropping neutral gum for sealing, observing under a microscope, and taking pictures.

### Ca^2+^ concentration detection

After C2C12 myoblasts were continuously induced to differentiate for 6 days, the concentration of Ca^2+^ was measured using a fluorescence microscope. In a 24‐well plate, PBS was used to wash the C2C12 cells twice, and the cells were incubated with the Ca^2+^ indicator of 10 μmoL/L Fluo‐3/AM at 37°C for 30 min. A fluorescence microscope was used to detect the fluorescence signals of all groups at an excitation wavelength of 488 nm and emission wavelength of 525 nm.

### Statistical analysis

In this study, all the experiments were divided into three parallel groups, all cell experiments were repeated three times, and animal experiments were repeated five times. Statistical analysis was performed using GraphPad Prism 8.0 (GraphPad Software Inc., San Diego, Calif., USA). The results are expressed as the mean ± standard deviation; a *t* test was used for comparison of differences between groups, and one‐way ANOVA was used for comparison of differences between multiple groups. *P* < 0.05 was considered statistically significant.

## Result

### Changes in Wnt5a and myogenic regulatory factors expression during myogenic differentiation and skeletal muscle regeneration

**Figure 1:**
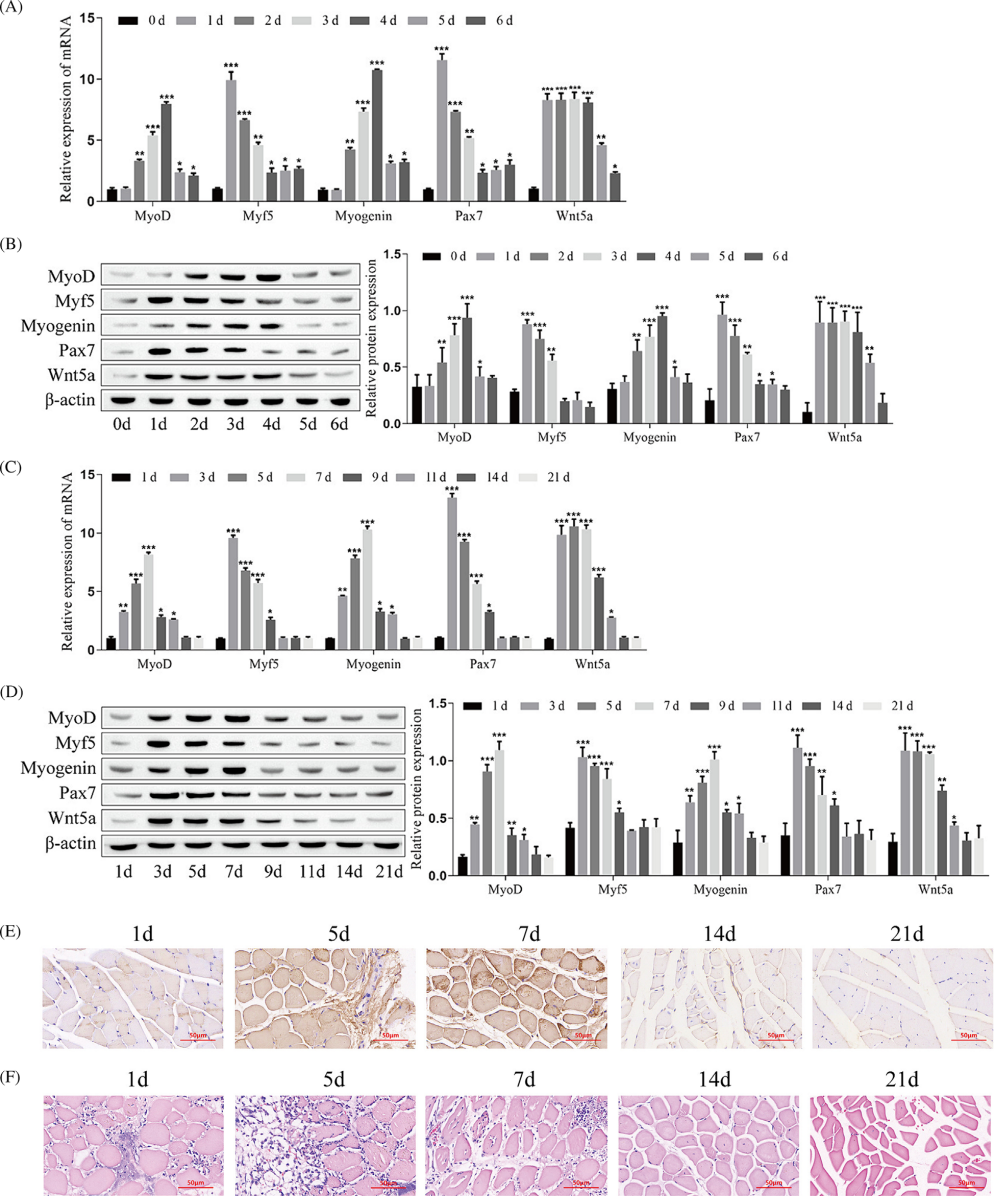
Expression of Wnt5a, Pax7, and MRFs during myogenic differentiation of C2C12 cells and muscle regeneration of mice. (A) Detecting the mRNA levels of MyoD, Myf5, Myogenin, Pax7, and Wnt5a in C2C12 myoblasts by RT–qPCR. (B) Detection of the protein levels of MyoD, Myf5, Myogenin, Pax7, and Wnt5a in C2C12 myoblasts by western blotting. (C) Detecting the mRNA levels of MyoD, Myf5, Myogenin, Pax7, and Wnt5a in the injured muscles of mice by RT–qPCR. (D) Detecting the protein levels of MyoD, Myf5, Myogenin, Pax7, and Wnt5a in the injured muscles of mice by western blotting. (E) Detection of MyoD expression in the injured muscle of mouse by immunohistochemistry. (F) Detecting histomorphological changes during repair after muscle injury in mice by HE staining. (Panels A, B: Compared with 0d, *P < 0.05, **P < 0.01, ***P < 0.001; panels C, D: Compared with 1d, *P < 0.05, **P < 0.01, ***P < 0.001).

First, C2C12 myoblasts were continuously induced to differentiate for 6 days to establish the model of myogenic differentiation. The results showed that C2C12 myoblasts showed progressive differentiation with increasing culture days (Figure S1A). RT‐qPCR and western blotting showed that the levels of Wnt5a, Pax7, MyoD, Myf5, and Myogenin were significantly upregulated. Among them, the expressions of Wnt5a, Myf5, and Pax7 reached their peak on the day 1, and MyoD and Myogenin peaked on the day 4 (Figure 1A,B).

Then, a model of acute skeletal muscle injury was established by injecting CTX into the tibialis anterior muscle in mice. During the repair process after skeletal muscle injury, RT‐qPCR and western blotting showed that the levels of Wnt5a, MyoD, Myf5, Pax7, and Myogenin were significantly increased, and the expression of MyoD and Myogenin peaked on the day 7, while Myf5, Pax7, and Wnt5a peaked on the day 3 (Figure 1C,D). Immunofluorescence results showed that the expression of MHC was significantly upregulated during repair after skeletal muscle injury and peaked at day 7 (Figure S1B). Immunohistochemical results showed that MyoD levels reached a maximum on day 7 after skeletal muscle injury in mice and then decreased (Figure 1E). HE staining results showed that the inflammatory cell infiltration in muscle tissue gradually increased on days 1–5 after CTX injection, and the morphology of cells in the damaged area changed and cells lost regular arrangement; on days 7–21 after CTX injection, the inflammatory cell infiltration gradually decreased, the nuclei moved toward the center of the cells, and the muscle fibres regained regular arrangement (Figure 1F). These results suggest that the levels of Wnt5a, MyoD, Myf5, Myogenin, and MHC are up‐regulated during the differentiation of myoblasts and the repair of skeletal muscle injury and participate in the formation of myotubes and mature muscle fibres.

### The role of Wnt5a in C2C12 myogenic differentiation

**Figure 2:**
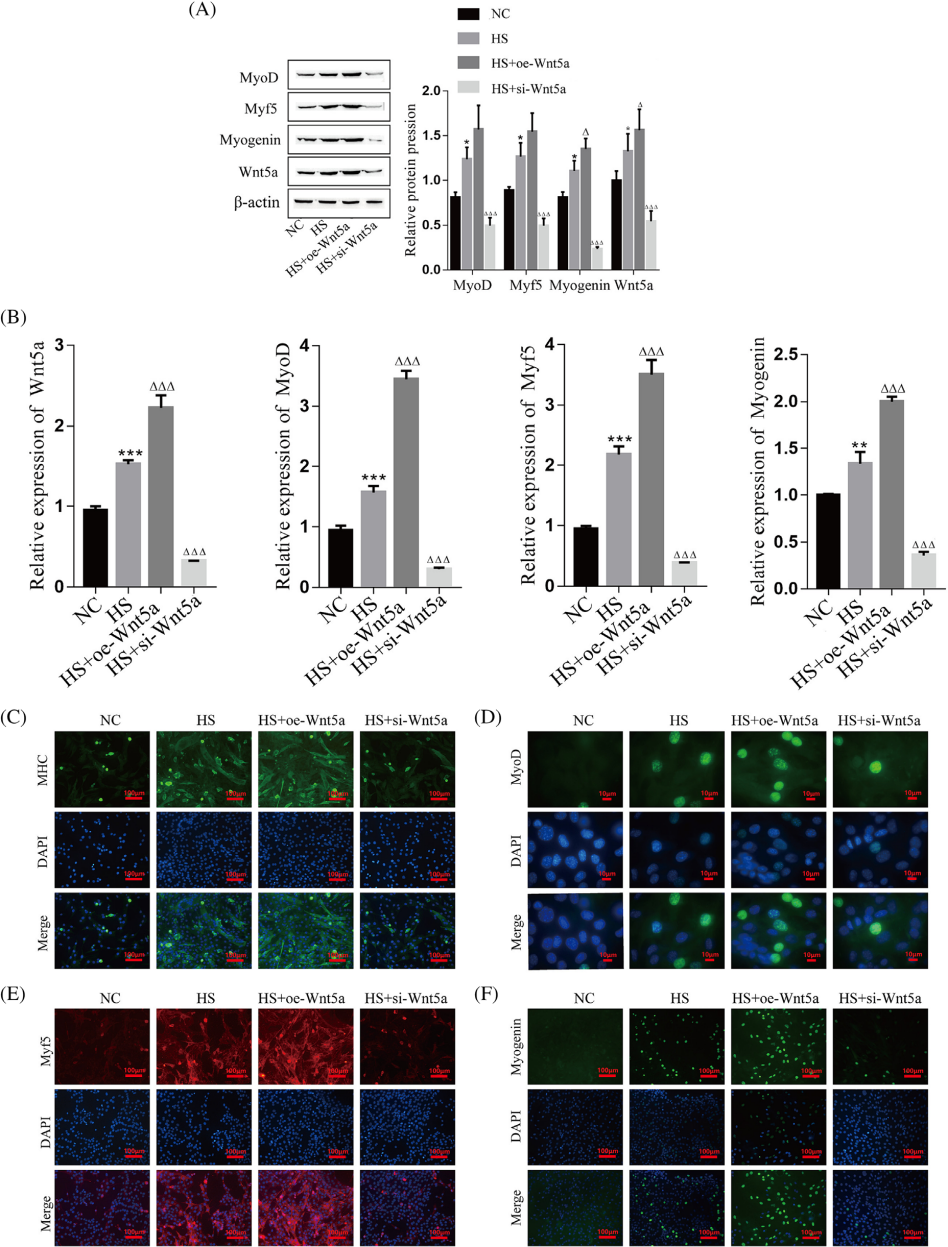
Role of Wnt5a in myogenic differentiation of C2C12 cells. (A) Detecting the protein expression of Wnt5a, MyoD, Myf5 and Myogenin by western blot. (B) Detecting the mRNA levels of Wnt5a, MyoD, Myf5 and Myogenin by RT‐qPCR. (C–F) Immunofluorescence was used to detect the expression of MHC (green), MyoD (green), Myf5 (red) and Myogenin (green), and nuclei were stained with DAPI (blue). Compared with the NC group, *P < 0.05, **P < 0.01, and ***P < 0.001; compared with the HS group, Δ*P* < 0.05, ΔΔΔ*P* < 0.001. C2C12 cells were continuously induced to differentiate for 4 days and subsequently sampled for analysis. HS, horse serum; NC, negative control.

We transfected C2C12 myoblasts with Wnt5a overexpression and knockdown lentiviral vectors to further explore the role of Wnt5a in myoblast differentiation (Figure S2A). The western blot assay showed that the Wnt5a protein expression level was significantly upregulated after overexpression but significantly downregulated after transfection with the Wnt5a knockdown vector (Figure S2B), indicating successful transfection of the lentiviral vector. During myogenic differentiation of C2C12 cells, the protein and mRNA expression of Wnt5a, MyoD, Myf5, and Myogenin was increased after overexpression of Wnt5a, while they were significantly decreased after knockdown of Wnt5a (Figure 2A,B). According to immunofluorescence, the expression of MyoD, Myf5, Myogenin, and MHC during myogenic differentiation could be promoted by the overexpression of Wnt5a, and their expression could be inhibited by knockdown of Wnt5a (Figure 2C–F), as well as Desmin (Figure S2C). These results suggest that Wnt5a promotes C2C12 myoblast differentiation and myotube formation by upregulating the levels of MyoD, Myf5, Myogenin, MHC, and Desmin.

### The role and mechanism of Wnt5a pathway‐mediated Ca^2+^ channel opening in C2C12 myoblast differentiation and skeletal muscle development

**Figure 3:**
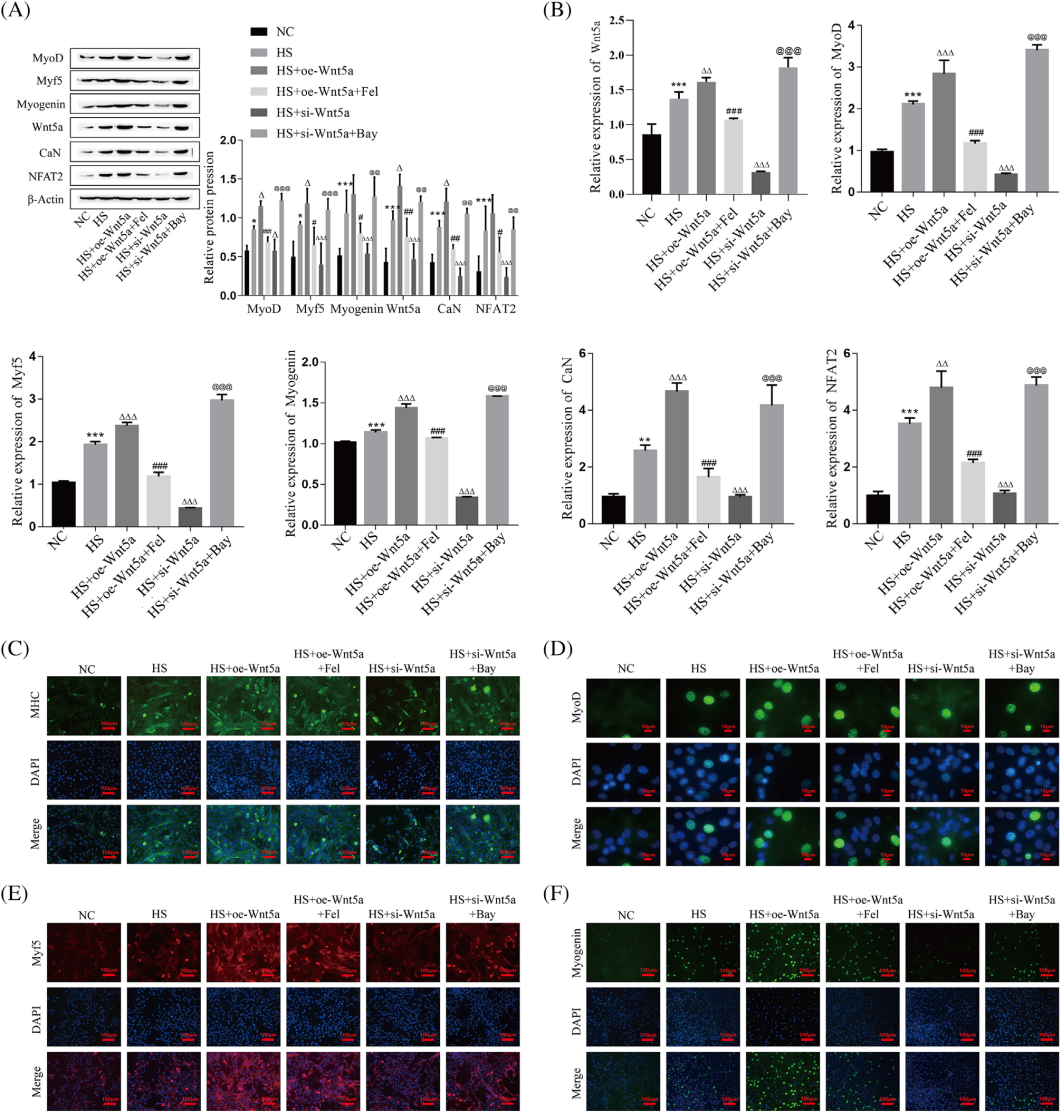
The role and mechanism of Wnt5a pathway‐mediated Ca^2+^ channel opening in myogenic differentiation of C2C12 cells. (A) The protein expression levels of Wnt5a, CaN, NFAT2, MyoD, Myf5, and Myogenin in C2C12 myoblasts were detected by western blot. (B) The mRNA expression levels of Wnt5a, CaN, NFAT2, MyoD, Myf5, and Myogenin in C2C12 myoblasts were detected by RT‐qPCR. (C–F) The expression levels of MHC (green), MyoD (green), Myf5 (red), and Myogenin (green) in C2C12 myoblasts were detected by immunofluorescence, and nuclei were stained with DAPI (blue). Compared with the NC group, *P < 0.05, **P < 0.01, ***P < 0.001; compared with the HS group, ^Δ^ *P* < 0.05, ^ΔΔ^ *P* < 0.01, ^ΔΔΔ^ *P* < 0.001; compared with the HS + oe‐Wnt5a group, ^#^ *P* < 0.05, ^##^ *P* < 0.01, ^###^ *P* < 0.001; compared with the HS + si‐Wnt5a group, ^@@^ *P* < 0.01, ^@@@^ *P* < 0.001. C2C12 cells were continuously induced to differentiate for 4 days and subsequently sampled for analysis. HS, horse serum; NC, negative control.

To confirm the role and mechanism of Wnt5a pathway‐mediated Ca^2+^ channel opening in C2C12 myoblast differentiation, we transfected C2C12 myoblasts with Wnt5a overexpression or knockdown lentiviral vectors and then used the Ca^2+^ channel inhibitor Fel or agonist Bay. The protein and mRNA expression levels of Wnt5a, CaN, NFAT2, MyoD, Myf5, and Myogenin were upregulated during myogenic differentiation of C2C12 cells after Wnt5a overexpression, and their expression levels were significantly downregulated after the addition of Ca^2+^ inhibitors on this basis; in contrast, the protein and mRNA expression of Wnt5a, CaN, NFAT2, MyoD, Myf5, and Myogenin were significantly decreased after Wnt5a knockdown, and their expression was significantly increased after the addition of Ca^2+^ agonists on this basis (Figure 3A,B). According to the results of immunofluorescence, the expression levels of MyoD, Myf5, Myogenin, and MHC were significantly increased after overexpression of Wnt5a, and their expression levels were significantly decreased after the addition of Ca^2+^ inhibitors on this basis. Conversely, knockdown of Wnt5a was followed by a significant decrease in MHC, MyoD, Myf5, and Myogenin expression levels, and their expression levels were significantly increased after the addition of Ca^2+^ agonists on this basis (Figure 3C–F).

At the same time, the expression changes of Desmin and NFAT2 during myogenic differentiation showed the same expression trends as those of MHC, MyoD, Myf5, and Myogenin (Figure S3A,B). In addition, we used fluorescence microscopy to observe the changes of Ca^2+^ expression during myogenic differentiation of C2C12 cells. The results showed that the Ca^2+^ concentration increased significantly after Wnt5a overexpression, and its expression decreased after adding Ca^2+^ inhibitor on this basis; on the contrary, Ca^2+^ concentration decreased significantly after Wnt5a knockdown, and its expression increased after adding Ca^2+^ agonist on this basis (Figure S3C). In addition, to verify that Wnt5a regulates MRFs by activating CaN, we added CaN inhibitor (CNI) based on overexpression of Wnt5a. The results revealed that CNI reversed the promotion of MyoD, Myf5, and Myogenin by Wnt5a (Figure S3D,E). The above results suggest that the opening or closing of Ca^2+^ channels can promote or inhibit the process of differentiation during myogenic differentiation of C2C12 cells, respectively.

**Figure 4:**
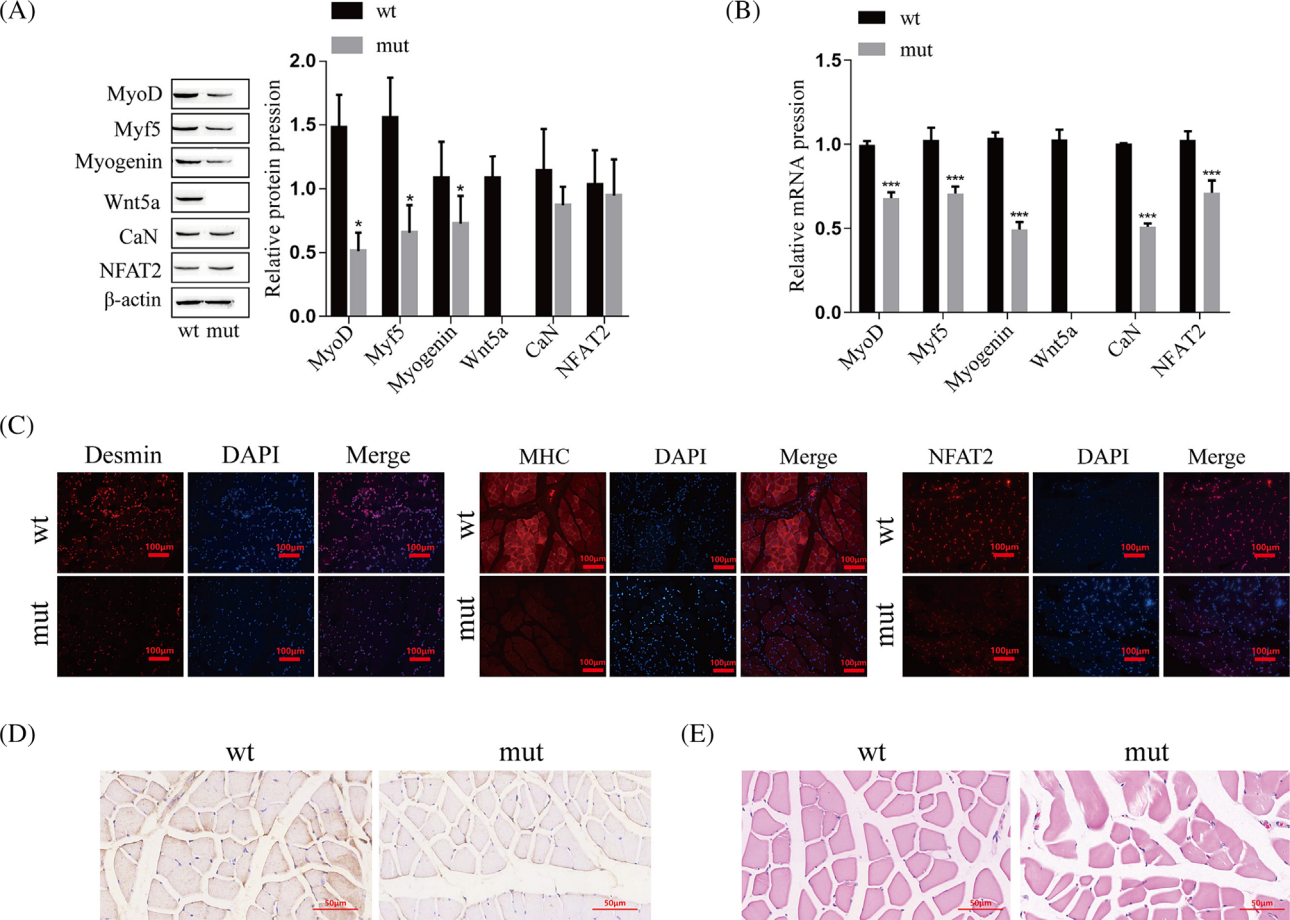
The role and mechanism of Wnt5a pathway in skeletal muscle development. (A) Detecting the protein levels of Wnt5a, CaN, NFAT2, MyoD, Myf5, and Myogenin in mouse skeletal muscle by western blotting. (B) Detecting the mRNA levels of Wnt5a, CaN, NFAT2, MyoD, Myf5, and Myogenin in mouse skeletal muscle by RT–qPCR. (C) Detection of the levels of Desmin (red), MHC (red), and NFAT2 (red) in mouse skeletal muscle by immunofluorescence, and the nuclei were stained with DAPI (blue). (D) The level of MyoD in mouse skeletal muscle was detected by immunohistochemistry. (E) Morphological changes in the mouse tibialis anterior muscle were observed by HE staining. Compared with the wt group, *P < 0.05, ***P < 0.001. HS, horse serum; Mut, Mutant type; NC, negative control; wt, wild‐type.

Moreover, to further explore the effects of Wnt5a on skeletal muscle development, we constructed the Wnt5a knockout mouse model. The results of western blot showed that, compared with wild type mice, the protein expression of MyoD, Myf5, and Myogenin in mutant mice was lower, and Wnt5a was not expressed, while the expression levels of CaN and NFAT2 were not significantly decreased (Figure 4A). RT‐qPCR results showed that in the skeletal muscle of Wnt5a knockout mice, Wnt5a mRNA was not expressed, and the mRNA levels of MyoD, Myf5, Myogenin, CaN, and NFAT2 were significantly decreased (Figure 4B). Then, immunofluorescence was used to detect the levels of Desmin, MHC, and NFAT2 in the skeletal muscle of mice, and the results showed that the expression of Desmin, MHC, and NFAT2 in mutant mice was lower than that in wild‐type mice (Figure 4C). Immunohistochemical results showed that MyoD was highly expressed in wild‐type mice and lowly expressed in mutant mice (Figure 4D). HE staining showed that the muscle fibres of tibialis anterior were tightly arranged in wild‐type mice, while those of mutant mice were loosely arranged (Figure 4E).

These results indicated that the Wnt5a/Ca^2+^/CaN/NFAT2 signalling pathway mediated the opening of Ca^2+^ channels, regulated the expression of CaN, NFAT2, MyoD, Myf5, Myogenin, MHC, and Desmin, and promoted the differentiation of C2C12 myoblasts and the development of skeletal muscle.

### The role and mechanism of curcumin in C2C12 myoblast differentiation and skeletal muscle regeneration

**Figure 5:**
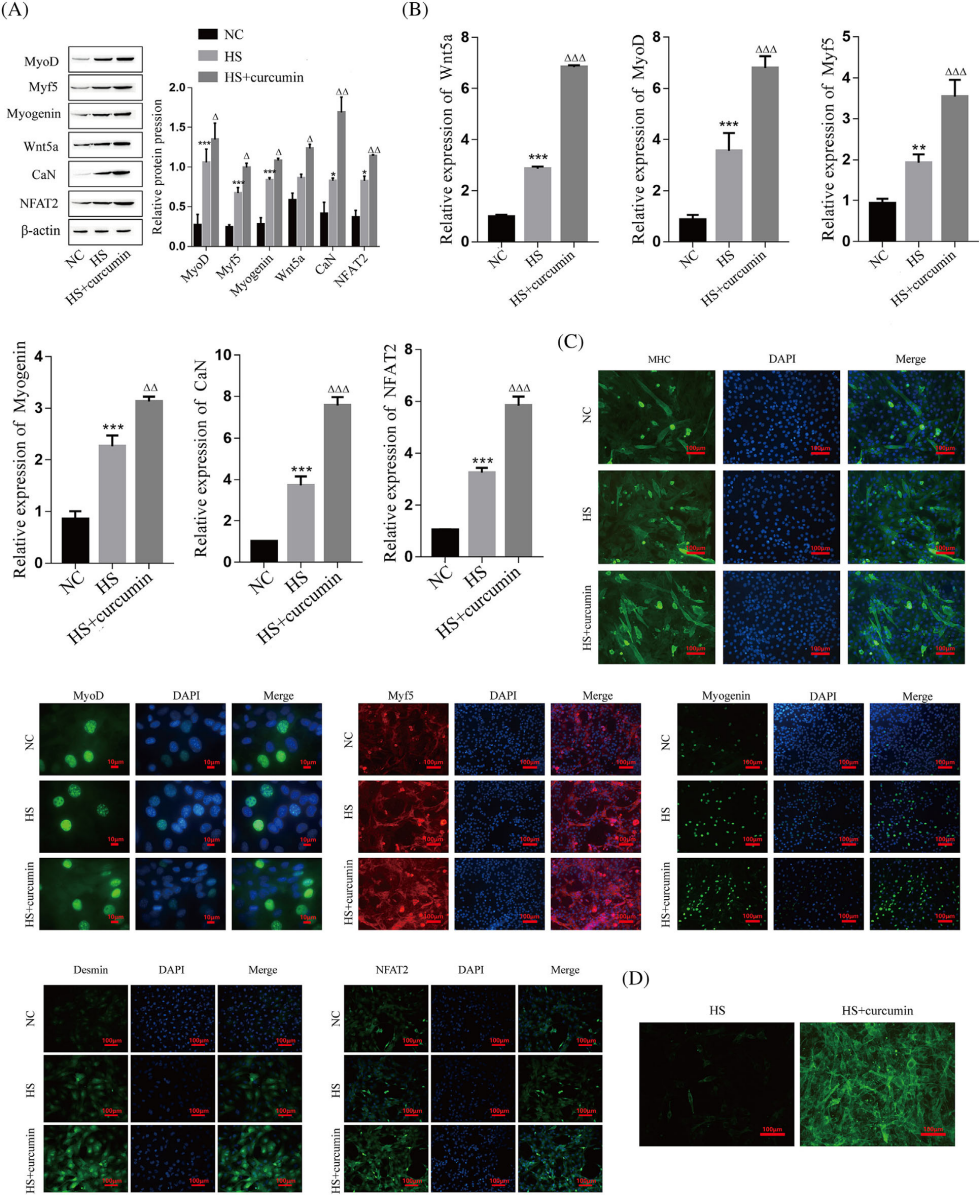
Effects and mechanisms of curcumin on myogenic differentiation of C2C12 cells. (A) Protein expression levels of Wnt5a, CaN, NFAT2, MyoD, Myf5, and Myogenin in C2C12 myoblasts were detected by western blot. (B) mRNA levels of Wnt5a, CaN, NFAT2, MyoD, Myf5, and Myogenin in C2C12 myoblasts were detected by RT–PCR. (C) The expression levels of MHC (green), MyoD (green), Myf5 (red), Myogenin (green), Desmin (green), and NFAT2 (green) were detected by immunofluorescence in C2C12 myoblasts, and the nuclei were stained with DAPI (blue). D: Ca^2+^ concentration of C2C12 myoblasts was detected by fluorescence microscopy. Compared with the NC group, *P < 0.05, **P < 0.01, ***P < 0.001; compared with the HS group, ^Δ^ *P* < 0.05, ^ΔΔ^ *P* < 0.01, ^ΔΔΔ^ *P* < 0.001. C2C12 cells were continuously induced to differentiate for 4 days and subsequently sampled for analysis. HS, horse serum; NC, negative control.

To investigate the role of curcumin in the myogenic differentiation of C2C12 cells, we treated C2C12 myoblasts with curcumin for 24 h. The western blot and RT–qPCR results showed that the protein and mRNA expression levels of Wnt5a, CaN, NFAT2, MyoD, Myf5, and Myogenin were significantly increased after curcumin treatment (Figure 5A,B). Immunofluorescence results showed that curcumin significantly promoted the expression levels of MHC, MyoD, Myf5, Myogenin, Desmin, and NFAT2 (Figure 5C). The concentration changes of Ca^2+^ during myogenic differentiation were detected by fluorescence microscopy, and the results showed that curcumin significantly promoted the intracellular Ca^2+^ level (Figure 5D).

**Figure 6:**
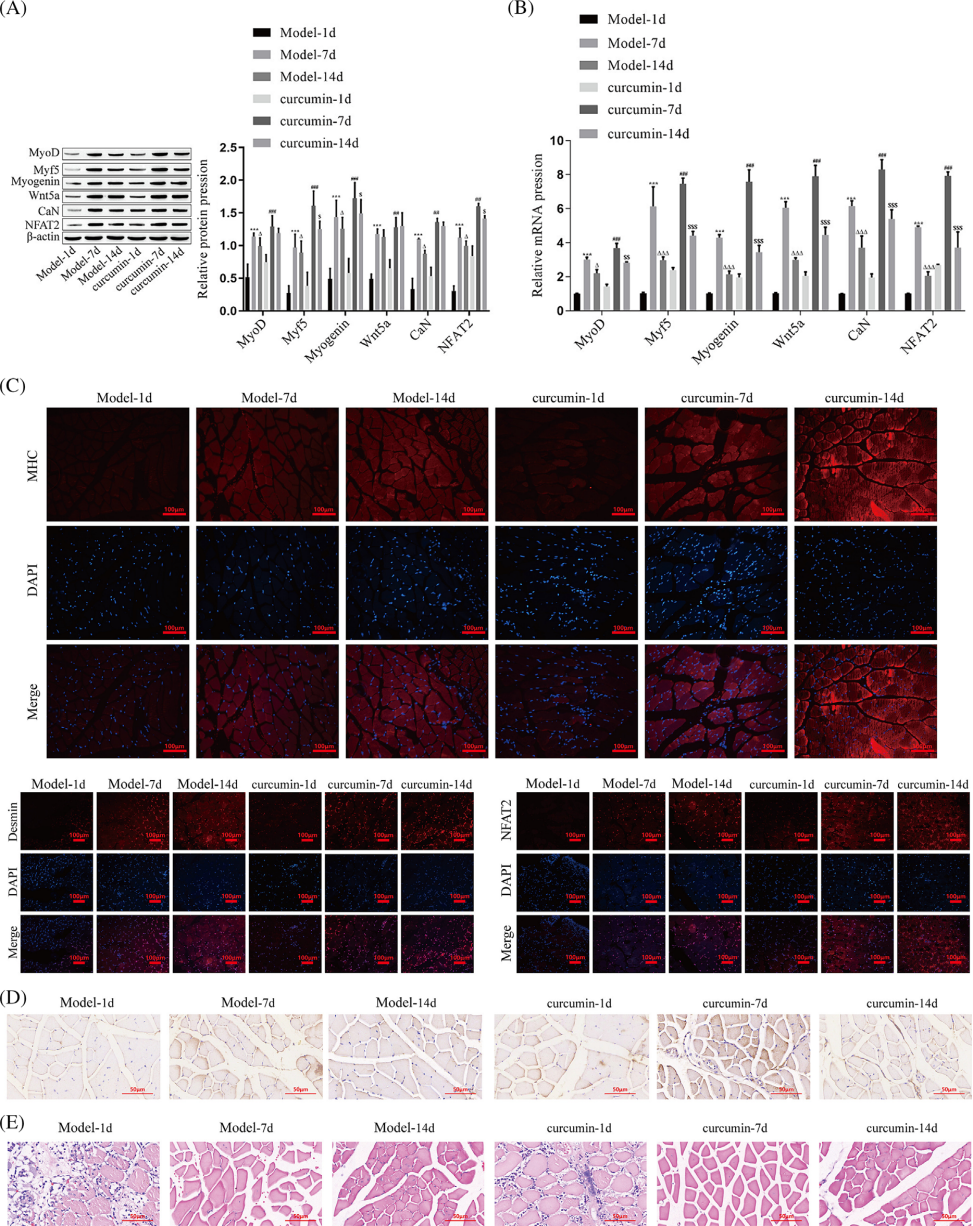
Effects and mechanisms of curcumin on skeletal muscle regeneration of mice. (A) Western blotting was performed to detect the protein levels of Wnt5a, CaN, NFAT2, MyoD, Myf5, and Myogenin. (B) RT–qPCR was performed to detect the mRNA levels of Wnt5a, CaN, NFAT2, MyoD, Myf5, and Myogenin. (C) The levels of MHC (red), Desmin (red), and NFAT2 (red) in the injured skeletal muscle of mice were detected by immunofluorescence, and the nuclei were stained with DAPI (blue). (D) The level of MyoD in the injured skeletal muscle of mice was detected by immunohistochemistry. (E) Histomorphological changes in injured skeletal muscle in mice were detected using HE staining. Compared with model‐1d, ***P < 0.001; compared with model‐7d, ^Δ^ *P* < 0.05, ^ΔΔΔ^ *P* < 0.001. Compared with curcumin‐1d, ^##^ *P* < 0.01, ^###^ *P* < 0.001; compared with curcumin‐7d, ^$^ *P* < 0.05, ^$$^ *P* < 0.01, ^$$$^ *P* < 0.001.

Moreover, we fed mice of skeletal muscle injury with curcumin for 14 days and detected the expression levels of related molecules on days 1, 7, and 14 after feeding. The results showed that the protein and mRNA expression levels of Wnt5a, CaN, NFAT2, MyoD, Myf5, and Myogenin increased significantly on day 7 but decreased on day 14, and the curcumin group had higher expression levels than the model group at the same time points (Figure 6A,B). According to the immunofluorescence results, the levels of MHC, Desmin, and NFAT2 at the site of skeletal muscle injury in mice continued to increase from 1 to 14 days after curcumin feeding (Figure 6C). Immunohistochemical results showed that MyoD levels in injured skeletal muscle of mice were highest on day 7 after curcumin feeding and then decreased. Compared with the model group, MyoD expression levels were higher in the curcumin group at the same time point (Figure 6D). In addition, we observed the effect of curcumin on the regeneration of skeletal muscle by HE staining. Compared with the model group, after 1 day of curcumin feeding, the degree of inflammatory cell infiltration in mouse muscle fibres was reduced; after 7 days, the regenerated muscle fibres were more regularly arranged, and the nuclei were centered; after 14 days, the repair and regeneration of injured muscles were basically completed, and the muscle fibres were closely arranged (Figure 6E).

The above results indicate that curcumin can accelerate myogenic differentiation of C2C12 cells and repair and regeneration of injured skeletal muscle in mice by increasing the levels of Wnt5a, CaN, NFAT2, MyoD, Myf5, Myogenin, MHC, and Desmin and promoting the opening of Ca^2+^ channels.

### Influences of curcumin on myoblast differentiation and skeletal muscle regeneration through the Wnt5a pathway

**Figure 7:**
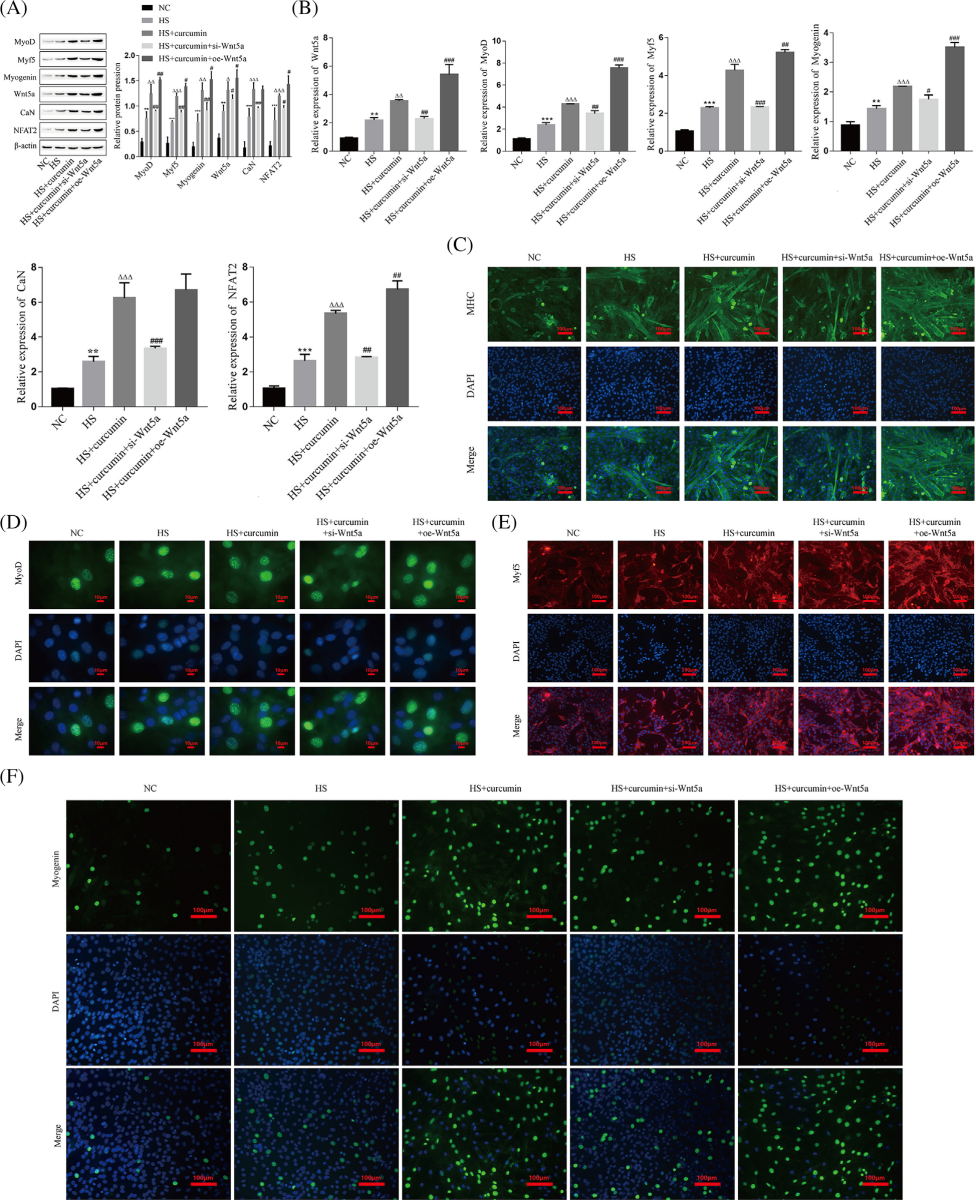
Curcumin affects myoblast differentiation via the Wnt5a pathway. (A) The protein expression levels of Wnt5a, CaN, NFAT2, MyoD, Myf5, and Myogenin in C2C12 myoblasts were detected by western blot. (B) The mRNA levels of Wnt5a, CaN, NFAT2, MyoD, Myf5, and Myogenin in C2C12 myoblasts were detected by RT–PCR. (C–F) The expression levels of MHC (green), MyoD (green), Myf5 (red), and Myogenin (green) in C2C12 myoblasts were detected by immunofluorescence, and the nuclei were stained with DAPI (blue). Compared with the NC group, **P < 0.01, ***P < 0.001; compared with the HS group, ^Δ^ *P* < 0.05, ^ΔΔ^ *P* < 0.01, ^ΔΔΔ^ *P* < 0.001; compared with the HS + curcumin group, ^#^ *P* < 0.05, ^##^ *P* < 0.01, ^###^ *P* < 0.001. C2C12 cells were continuously induced to differentiate for 4 days and ubsequently sampled for analysis. HS, horse serum; NC, negative control.

To further explore the specific mechanisms by which curcumin affects myoblast differentiation, we knocked down or overexpressed Wnt5a in curcumin‐treated C2C12 cells. The western blot and RT–qPCR results showed that curcumin treatment significantly upregulated the protein and mRNA levels of Wnt5a, CaN, NFAT2, MyoD, Myf5, and Myogenin, and knockdown of Wnt5a on this basis significantly inhibited the effect of curcumin, while overexpression of Wnt5a on this basis further promoted the expression of Wnt5a, NFAT2, MyoD, Myf5, and Myogenin, but had no significant effect on CaN (Figure 7A,B). According to the immunofluorescence results, knockdown of Wnt5a on the basis of curcumin treatment resulted in decreased levels of MyoD, Myf5, Myogenin, and MHC, while overexpression of Wnt5a on the basis of curcumin treatment further increased their expression levels (Figure 7C–F).

**Figure 8:**
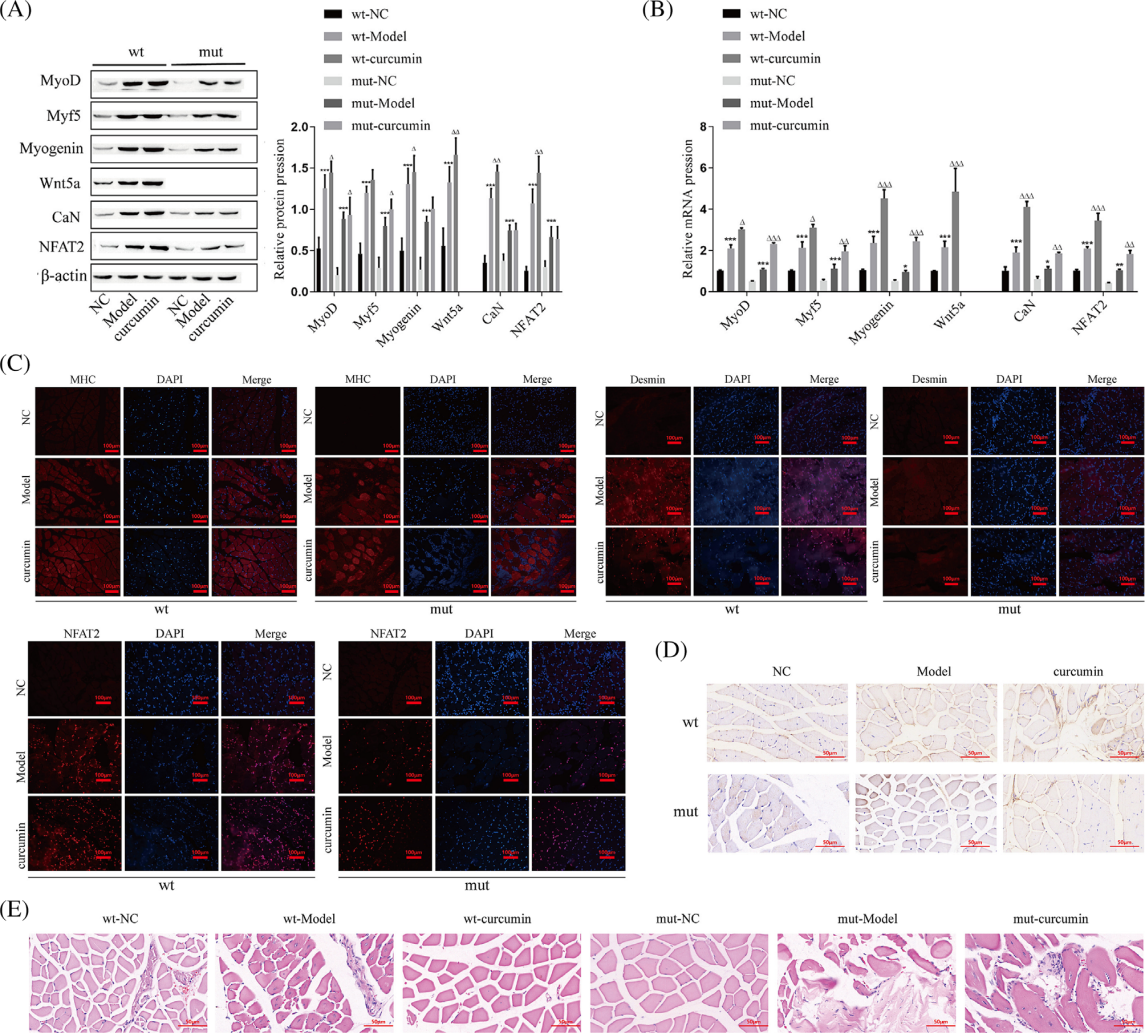
Curcumin affects skeletal muscle regeneration via the Wnt5a pathway. (A) The protein levels of MyoD, Myf5, Myogenin, Wnt5a, CaN, and NFAT2 were detected by western blotting. (B) The mRNA levels of MyoD, Myf5, Myogenin, Wnt5a, CaN, and NFAT2 were detected by RT–qPCR. (C) Immunofluorescence for the expression of MHC (red), Desmin (red), and NFAT2 (red) at the site of skeletal muscle injury in mice, and the nuclei were stained with DAPI (blue). (D) Immunohistochemistry was used to detect the level of MyoD at the site of skeletal muscle injury in mice. (E) Histomorphological changes in mice skeletal muscle after injury were observed by HE staining. Wt‐model compared with wt‐NC, ***P < 0.001; wt‐curcumin compared with wt‐model, ^Δ^ *P* < 0.05, ^ΔΔ^ *P* < 0.01, ^ΔΔΔ^ *P* < 0.001. Mut‐model compared with Mut‐NC, *P < 0.05, **P < 0.01, ***P < 0.001; Mut‐curcumin compared with mut‐model, ^Δ^ *P* < 0.05, ^ΔΔ^ *P* < 0.01, ^ΔΔΔ^ *P* < 0.001. Mut, mutant type; NC, negative control; wt, wild type.

Meanwhile, we fed Wnt5a knockout mice with curcumin to further explore the effect of curcumin on skeletal muscle regeneration. Next, the protein and mRNA expression of Wnt5a, CaN, NFAT2, MyoD, Myf5, and Myogenin were examined in the injured skeletal muscle of wild‐type mice and Wnt5a knockout mutant mice. The results showed that Wnt5a was not expressed in the skeletal muscle of Wnt5a mutant mice, and for MyoD, Myf5, Myogenin, CaN, and NFAT2, the mutant mice expressed lower levels than the wild‐type mice in both the model and curcumin groups (Figure 8A,B). Immunofluorescence results showed that the expression of MHC, NFAT2, and Desmin was less increased in the Wnt5a mutant mouse model group and curcumin group than in wild‐type mice (Figure 8C). Immunohistochemical results showed that knockdown of the Wnt5a gene also resulted in lower expression of MyoD in the model and curcumin groups of mutant mice than in wild‐type mice (Figure 8D). The histomorphological changes at the site of skeletal muscle injury in mice were observed by HE staining, and the results showed that compared with wild‐type mice, mutant mice formed fewer regenerated muscle fibres after curcumin administration, the muscle fibres were loosely arranged, and only a small amount of muscle tissue was repaired and regenerated (Figure 8E).

The above results suggest that curcumin affects myogenic differentiation and skeletal muscle regeneration through activation of the Wnt5a/Ca^2+^/CaN/NFAT2 pathway and that deletion of Wnt5a hinders the process of myogenic differentiation and muscle regeneration.

## Discussion

Currently, the molecular mechanisms of myoblast differentiation and muscle regeneration have been extensively studied. After muscle fibre damage, quiescent satellite cells can proliferate and be activated to enter the cell cycle.^21^ Activated satellite cells become myoblasts, which are characterized by high expression of MyoD and Myf5.^22^ In muscle regeneration, MyoD and Myf5 have different roles, and MyoD can promote the activation of satellite cells,^23^ while Myf5 promotes satellite cell self‐renewal.^24^ In addition, terminal differentiation of myoblasts is characterized by upregulation of myogenin and MRF4,^25^ but MRF4 has a smaller role in myogenic differentiation.^26^ In addition, MHC expression is a hallmark of myotube formation,^5^ and Desmin, as a muscle‐specific intermediate filament protein, plays an important role in muscle structure and force transmission.^27^ Therefore, detecting changes in the expression of MyoD, Myf5, Myogenin, MHC, and Desmin is important to understand the progression of myogenic differentiation. Our results confirm that the expression of MyoD, Myf5, Myogenin, MHC, and Desmin is upregulated during myogenic differentiation of C2C12 cells and repair of injured skeletal muscle and is involved in myotube formation and muscle regeneration. In addition, we observed that Wnt5a overexpression or knockdown did upregulate or downregulate the expression levels of MyoD, Myf5, Myogenin, MHC, and Desmin, respectively. This suggests that Wnt5a affects C2C12 myoblast differentiation and skeletal muscle regeneration processes by influencing the expression of MyoD, Myf5, Myogenin, MHC, and Desmin.

In skeletal muscle, Ca^2+^ is mainly stored in the sarcoplasmic reticulum of myocytes, where it triggers muscle contraction by excitation‐contraction coupling. However, the role of Ca^2+^ not only lies in influencing muscle relaxation and contraction but also plays an important role in muscle development, maintenance, and regeneration.^11^ In myoblasts, Ca^2+^ is involved in differentiation and fusion. Ca^2+^ influx into myoblasts is a prerequisite for myotube formation,^28^ and myoblast fusion to form myotubes is mediated through Ca^2+^ channels.^29^ Regarding Ca^2+^ channels, studies have shown that sarcoplasmic reticulum stored Ca^2+^ can be released into the cytoplasm via ryanodine receptors^30^ and inositol triphosphate receptors,^31^ thereby triggering a series of physiological effects. In addition, extracellular Ca^2+^ can also enter the cell via Ca^2+^ channels on the cell membrane, such as transient receptor potential channel 3 (TRPC3).^32^ In this study, we found that overexpression or knockdown of Wnt5a could induce an increase or decrease in intracellular Ca^2+^ concentration, respectively, while Ca^2+^ channel opening or closing could promote or inhibit the expression levels of CaN, NFAT2, MRFs, MHC, and Desmin, respectively. This suggests that the Wnt5a/Ca^2+^/CaN/NFAT2 signalling pathway mediates Ca^2+^ channel opening to promote myoblast differentiation and skeletal muscle regeneration. However, the channels through which Ca^2+^ enters the cytoplasm and whether Ca^2+^ originates from the sarcoplasmic reticulum or extracellular space were not discussed in this study.

Currently, experimental studies of curcumin have yielded several favourable results in terms of antioxidant, anti‐inflammatory, and anticancer properties.^33^ As research into the medicinal value of curcumin deepened, it was discovered that curcumin also has a protective effect against muscle damage. For example, curcumin increased the number of satellite cells in a muscle atrophy model,^34^ increased the diameter of myotubes in injured muscles, reduced muscle fibrosis and inflammation, and thus promoted muscle regeneration.^35^ However, the specific molecular mechanisms by which curcumin promotes the regeneration of injured muscles are not well understood. We found that curcumin treatment promoted the expression of Wnt5a, CaN, NFAT2, MyoD, Myf5, Myogenin, MHC, and Desmin and contributed to an increase in intracellular Ca^2+^ concentration. Meanwhile, HE staining showed that curcumin accelerated the repair and regeneration of injured skeletal muscle in mice. In addition, knockdown of Wnt5a on the basis of curcumin intervention inhibited the positive effect of curcumin on myogenic differentiation of C2C12 cells, and the effect of curcumin on skeletal muscle regeneration was also greatly reduced in Wnt5a knockout mice. This evidence suggests that curcumin accelerates myogenic differentiation and muscle regeneration by activating the Wnt5a/Ca^2+^/CaN/NFAT2 signalling pathway and promoting the expression of MRFs, MHC, and Desmin.

Our study has several limitations. First, we demonstrated that curcumin affects myogenic differentiation and muscle regeneration by activating the Wnt5a pathway and mediating Ca^2+^ channel opening. However, it is not clear what channel curcumin opens to trigger Ca^2+^ influx or release. Previous studies have shown that TRPC3 is upregulated during myogenic differentiation of C2C12 cells,^36^ suggesting that TRPC3 may be involved in the process of myogenic differentiation. Moreover, TPRC3 can mediate the inward flow of extracellular Ca^2+^ to the cytoplasm.^32^ Therefore, we speculate that exogenous Ca^2+^ may be involved in the process of myogenic differentiation and muscle regeneration; however, the specific mechanism needs to be further experimentally explored by our research team. Second, we did not assay for changes in other substances after curcumin treatment of cells and mice. There may be multiple molecular mechanisms by which curcumin promotes muscle regeneration. Therefore, we do not exclude the possibility that changes in other substances may have an effect on myoblast differentiation and repair and regeneration of injured skeletal muscle. Third, we did not set up a control group of uninjured mice supplemented with curcumin, because we were mainly concerned with the promotion of curcumin on the regeneration of injured skeletal muscle. Moreover, previous studies have shown that curcumin does not induce embryonic MHC expression in undamaged skeletal muscle^15^ and that it does not promote myogenic differentiation of normal myoblasts (undamaged).^37^ Despite these limitations, our study demonstrated the protective effect of curcumin on skeletal muscle and its possible mechanism, which will provide reliable theoretical support for the clinical treatment of skeletal muscle injury.

## Conclusions

The Wnt5a/Ca^2+^/CaN/NFAT2 signalling pathway plays an important role in the myogenic differentiation of C2C12 cells and the repair and regeneration of injured skeletal muscle. By promoting the expression of Wnt5a, curcumin activates the Wnt5a signalling pathway and promotes the expression of myogenic regulatory factors, which in turn promotes myogenic differentiation and skeletal muscle regeneration.

### Mechanism diagram

As shown in Figure S4, Wnt5a is produced by cells and released to the outside of the cell, and curcumin stimulates cells to induce high Wnt5a expression. Subsequently, Wnt5a binds to receptors on cell membranes. Then, through a series of cascade reactions, Ca^2+^ channels on the endoplasmic reticulum (RyR or IP3R) or cell membranes (TRPC3) are opened, allowing Ca^2+^ to flow into the cytoplasm, which in turn activates CaN expression. CaN induces NFAT2 dephosphorylation and translocation to the nucleus, thus promoting the expression of downstream factors MyoD, Myf5, Myogenin, MHC, and Desmin, which in turn accelerates myogenic differentiation of C2C12 cells and regeneration of injured skeletal muscle. (Note: The dashed part is speculative for this study, and further experiments are needed to justify it.).

## Funding

This study is supported by the National Natural Science Foundation of China (grant numbers 82060420) and the Natural Science Foundation of Jiangxi Province, China (grant numbers 20212BAB206004). The funders have no role in study design, data collection or analysis, preparation of the manuscript, or the decision to publish. The contents of this publication are solely the responsibility of authors and do not necessarily represent the official views of the funding sources.

## Conflict of interest

The authors report no conflict of interest.

## Supporting information


**Table S1.** The primer sequence of the genes.
**Figure S1.** Cell culture and expression of MHC during muscle regeneration of mice.
**Figure S2.** Role of Wnt5a in myogenic differentiation of C2C12 cells.
**Figure S3.** The role and mechanism of Wnt5a pathway‐mediated Ca^2+^ channel opening in C2C12 myoblast differentiation.
**Figure S4.** Schematic diagram of curcumin activation of the Wnt5a signalling pathway to promote myogenic differentiation and muscle regeneration.

## Data Availability

The datasets generated during the current study are available from the corresponding author on reasonable request.

## References

[1] Karalaki M , Fili S , Philippou A , Koutsilieris M . Muscle regeneration: cellular and molecular events. In Vivo 2009;23:779–796. 19779115

[2] Gonzalez ML , Busse NI , Waits CM , Johnson SE . Satellite cells and their regulation in livestock. J Anim Sci 2020;98. 10.1093/jas/skaa081 PMC7193651 32175577

[3] Bachman JF , Klose A , Liu W , Paris ND , Blanc RS , Schmalz M , et al. Prepubertal skeletal muscle growth requires Pax7‐expressing satellite cell‐derived myonuclear contribution. Development (Cambridge, England) 2018;145. 10.1242/dev.167197 PMC6215399 30305290

[4] Asfour HA , Allouh MZ , Said RS . Myogenic regulatory factors: the orchestrators of myogenesis after 30 years of discovery. In Experimental biology and medicine, Vol. 243. Maywood N.J.; 2018. p 118–128. 29307280 10.1177/1535370217749494 PMC5788151

[5] Sato H , Funaki A , Kimura Y , Sumitomo M , Yoshida H , Fukata H , et al. Ethanol extract of *Cyclolepis genistoides* D. Don (palo azul) induces formation of myotubes, which involves differentiation of C2C12 myoblast cells. Nutr Res (New York, NY) 2016;36:731–741. 10.1016/j.nutres.2016.02.011 27262535

[6] Agnetti G , Herrmann H , Cohen S . New roles for desmin in the maintenance of muscle homeostasis. FEBS J 2022;289:2755–2770. 33825342 10.1111/febs.15864

[7] Paluszczak J . The significance of the dysregulation of canonical Wnt signaling in head and neck squamous cell carcinomas. Cells 2020;9:723. 32183420 10.3390/cells9030723 PMC7140616

[8] Suryawanshi A , Hussein MS , Prasad PD , Manicassamy S . Wnt signaling cascade in dendritic cells and regulation of anti‐tumor immunity. Front Immunol 2020;11:122. 32132993 10.3389/fimmu.2020.00122 PMC7039855

[9] Girardi F , le Grand F . Wnt signaling in skeletal muscle development and regeneration. Prog Mol Biol Transl Sci 2018;153:157–179. 29389515 10.1016/bs.pmbts.2017.11.026

[10] Zhang ZK , Li J , Guan D , Liang C , Zhuo Z , Liu J , et al. A newly identified lncRNA MAR1 acts as a miR‐487b sponge to promote skeletal muscle differentiation and regeneration. J Cachexia Sarcopenia Muscle 2018;9:613–626. 29512357 10.1002/jcsm.12281 PMC5989759

[11] Tu MK , Levin JB , Hamilton AM , Borodinsky LN . Calcium signaling in skeletal muscle development, maintenance and regeneration. Cell Calcium 2016;59:91–97. 26944205 10.1016/j.ceca.2016.02.005 PMC4834241

[12] Shu Y , Xiang M , Zhang P , Qi G , He F , Zhang Q , et al. Wnt‐5a promotes neural development and differentiation by regulating CDK5 via Ca2+/calpain pathway. Cell Physiol Biochem 2018;51:2604–2615. 30562750 10.1159/000495932

[13] Farrera‐Hernández A , Marín‐Llera JC , Chimal‐Monroy J . WNT5A‐Ca(2+)‐CaN‐NFAT signalling plays a permissive role during cartilage differentiation in embryonic chick digit development. Dev Biol 2021;469:86–95. 33058830 10.1016/j.ydbio.2020.10.003

[14] Daou N , Lecolle S , Lefebvre S , Della Gaspera B , Charbonnier F , Chanoine C , et al. A new role for the calcineurin/NFAT pathway in neonatal myosin heavy chain expression via the NFATc2/MyoD complex during mouse myogenesis. Development 2013;140:4914–4925. 24301466 10.1242/dev.097428

[15] Thaloor D , Miller KJ , Gephart J , Mitchell PO , Pavlath GK . Systemic administration of the NF‐kappaB inhibitor curcumin stimulates muscle regeneration after traumatic injury. Am J Physiol 1999;277:C320–C329. 10444409 10.1152/ajpcell.1999.277.2.C320

[16] Yu T , Dohl J , Wang L , Chen Y , Gasier HG , Deuster PA . Curcumin ameliorates heat‐induced injury through NADPH oxidase‐dependent redox Signaling and mitochondrial preservation in C2C12 myoblasts and mouse skeletal muscle. J Nutr 2020;150:2257–2267. 32692359 10.1093/jn/nxaa201 PMC7919340

[17] Zhou J , Wu N , Lin L . Curcumin suppresses apoptosis and inflammation in hypoxia/reperfusion‐exposed neurons via Wnt signaling pathway. Med Sci Monitor 2020;26:e920445. 10.12659/MSM.920445 PMC7061587 32107363

[18] Ho C , Hsu YC , Lei CC , Mau SC , Shih YH , Lin CL . Curcumin rescues diabetic renal fibrosis by targeting superoxide‐mediated Wnt signaling pathways. Am J Med Sci 2016;351:286–295. 26992258 10.1016/j.amjms.2015.12.017

[19] Du J , Zhang Y , Shen L , Luo J , Lei H , Zhang P , et al. Effect of miR‐143‐3p on C2C12 myoblast differentiation. Biosci Biotechnol Biochem 2016;80:706–711. 26854366 10.1080/09168451.2015.1123604

[20] Peng S , Song C , Li H , Cao X , Ma Y , Wang X , et al. Circular RNA SNX29 sponges miR‐744 to regulate proliferation and differentiation of myoblasts by activating the Wnt5a/Ca(2+) signaling pathway. Mol Ther Nucleic Acids 2019;16:481–493. 31051333 10.1016/j.omtn.2019.03.009 PMC6495097

[21] Ahmad K , Shaikh S , Ahmad SS , Lee EJ , Choi I . Cross‐talk between extracellular matrix and skeletal muscle: implications for myopathies. Front Pharmacol 2020;11:142. 32184725 10.3389/fphar.2020.00142 PMC7058629

[22] Zevolis E , Philippou A , Moustogiannis A , Chatzigeorgiou A , Koutsilieris M . The effects of mechanical loading variations on the hypertrophic, anti‐apoptotic, and anti‐inflammatory responses of differentiated cardiomyocyte‐like H9C2 cells. Cells 2022;11. 10.3390/cells11030473 PMC8834179 35159283

[23] Manoharan P , Song T , Radzyukevich TL , Sadayappan S , Lingrel JB , Heiny JA . KLF2 in myeloid lineage cells regulates the innate immune response during skeletal muscle injury and regeneration. iScience 2019;17:334–346. 31326700 10.1016/j.isci.2019.07.009 PMC6652133

[24] de Almeida Mallmann B , Martin EM , Soo Kim K , Calderon‐Apodaca NL , Baxter MFA , Latorre JD , et al. Evaluation of bone marrow adipose tissue and bone mineralization on broiler chickens affected by wooden breast myopathy. Front Physiol 2019;10:674. 31191361 10.3389/fphys.2019.00674 PMC6549442

[25] Wu CL , Harasymowicz NS , Klimak MA , Collins KH , Guilak F . The role of macrophages in osteoarthritis and cartilage repair. Osteoarthr Cartil 2020;28:544–554. 10.1016/j.joca.2019.12.007 PMC7214213 31926267

[26] Zammit PS . Function of the myogenic regulatory factors Myf5, MyoD, Myogenin and MRF4 in skeletal muscle, satellite cells and regenerative myogenesis. Semin Cell Dev Biol 2017;72:19–32. 29127046 10.1016/j.semcdb.2017.11.011

[27] Kayman Kürekçi G , Kural Mangit E , Koyunlar C , Unsal S , Saglam B , Ergin B , et al. Knockout of zebrafish desmin genes does not cause skeletal muscle degeneration but alters calcium flux. Sci Rep 2021;11:7505. 33820917 10.1038/s41598-021-86974-w PMC8021586

[28] Bijlenga P , Liu JH , Espinos E , Haenggeli CA , Fischer‐Lougheed J , Bader CR , et al. T‐type alpha 1H Ca2+ channels are involved in Ca2+ signaling during terminal differentiation (fusion) of human myoblasts. Proc Natl Acad Sci U S A 2000;97:7627–7632. 10861024 10.1073/pnas.97.13.7627 PMC16596

[29] Benavides Damm T , Egli M . Calcium's role in mechanotransduction during muscle development. Cell Physiol Biochem 2014;33:249–272. 24525559 10.1159/000356667

[30] Levin JB , Borodinsky LN . Injury‐induced Erk1/2 signaling tissue‐specifically interacts with Ca(2+) activity and is necessary for regeneration of spinal cord and skeletal muscle. Cell Calcium 2022;102:102540. 35074688 10.1016/j.ceca.2022.102540 PMC9542431

[31] Toprak U , Doğan C , Hegedus D . A comparative perspective on functionally‐related, intracellular calcium channels: The insect ryanodine and inositol 1,4,5‐trisphosphate receptors. Biomolecules 2021;11:1031. 34356655 10.3390/biom11071031 PMC8301844

[32] Kim MS , Lee KP , Yang D , Shin DM , Abramowitz J , Kiyonaka S , et al. Genetic and pharmacologic inhibition of the Ca2+ influx channel TRPC3 protects secretory epithelia from Ca2+−dependent toxicity. Gastroenterology 2011;140:2115.e2101–2115.e2104. 10.1053/j.gastro.2011.02.052 PMC3109139 21354153

[33] Unlu A , Nayir E , Dogukan Kalenderoglu M , Kirca O , Ozdogan M . Curcumin (turmeric) and cancer. J BUON 2016;21:1050–1060. 27837604

[34] Mañas‐García L , Guitart M , Duran X , Barreiro E . Satellite cells and markers of muscle regeneration during unloading and reloading: effects of treatment with resveratrol and curcumin. Nutrients 2020;12:1870. 32585875 10.3390/nu12061870 PMC7353305

[35] Mahdy MAA , Akl MA , Madkour FA . Effect of chitosan and curcumin nanoparticles against skeletal muscle fibrosis at early regenerative stage of glycerol‐injured rat muscles. BMC Musculoskelet Disord 2022;23:670. 35836166 10.1186/s12891-022-05633-x PMC9281067

[36] Cheung KK , Yeung SS , Au SW , Lam LS , Dai ZQ , Li YH , et al. Expression and association of TRPC1 with TRPC3 during skeletal myogenesis. Muscle Nerve 2011;44:358–365. 21996795 10.1002/mus.22060

[37] Berger F , Büchsler I , Munz B . The effect of the NF‐kappa B inhibitors curcumin and lactacystin on myogenic differentiation of rhabdomyosarcoma cells. Differentiation Res Biol Divers 2012;83:271–281. 10.1016/j.diff.2012.02.001 22469857

