# Supplementary material for: Curcumin‐activated Wnt5a pathway mediates Ca2+ channel opening to affect myoblast differentiation and skeletal muscle regeneration
Source: J Cachexia Sarcopenia Muscle. 2024 Jul 10;15(5):1834–49. doi: 10.1002/jcsm.13535 (PMC11446719; doi:10.1002/jcsm.13535)
Supplement: Supplementary file 1 — Table S1. The primer sequence of the genes. Figure S1. Cell culture and expression of MHC during muscle regeneration of mice. Figure S2. Role of Wnt5a in myogenic differentiation of C2C12 cells. Figure S3. The role and mechanism of Wnt5a pathway‐mediated Ca2+ channel opening in C2C12 myoblast differentiation. Figure S4. Schematic diagram of curcumin activation of the Wnt5a signalling pathway to promote myogenic differentiation and muscle regeneration. [file JCSM-15-1834-s001.docx]

**Supplementary materials for *Curcumin-activated Wnt5a pathway mediates Ca^2+^ channel opening to affect myoblast differentiation and skeletal muscle regeneration***

**Supplementary** **Table 1**. The primer sequence of the genes.

**Supplementary Figure 1.** Cell culture and expression of MHC during muscle regeneration of mice.

**Supplementary Figure 2.** Role of Wnt5a in myogenic differentiation of C2C12 cells.

**Supplementary Figure 3.** The role and mechanism of Wnt5a pathway-mediated Ca^2+^ channel opening in C2C12 myoblast differentiation.

**Supplementary Figure 4.** Schematic diagram of curcumin activation of the Wnt5a signaling pathway to promote myogenic differentiation and muscle regeneration.

**Supplementary Table 1**. The primer sequence of the genes.

| Genes | Forward Primer | Reverse Primer |
| --- | --- | --- |
| Wnt5a | 5’-CTACCGCTTCGCCAAGGAGTTC-3’ | 5’-TAGCCACGCCCACAGCACAT-3’ |
| CaN | 5’-ATCTGCTCCGACGATGAACTGG-3’ | 5’-CTGTCCGTGCCGTTAGTCTCTG-3’ |
| NFAT2 | 5’-GGTGAGGCTGGTCTTCCGAGTT-3’ | 5’-GCTGTCTGTGCTCTGCTTCTCC-3’ |
| MyoD | 5’-CGCAAGACCACCAACGCTGAT-3’ | 5’-GCATCTGAGTCGCCACTGTAGT-3’ |
| Myf5 | 5’-ATGACGGACGGCTGCCAGTT-3’ | 5’-GTGCTCCGAAGGCTGCTACTCT-3’ |
| Myogenin | 5’-AGAGGAAGTCTGTGTCGGTGGA-3’ | 5’-GGTTTCGTCTGGGAAGGCAACA-3’ |
| β-actin | 5’-TGTGCTGTCCCTGTATGCCTCT-3’ | 5’-GGAACCGCTCGTTGCCAATAGT-3’ |

**
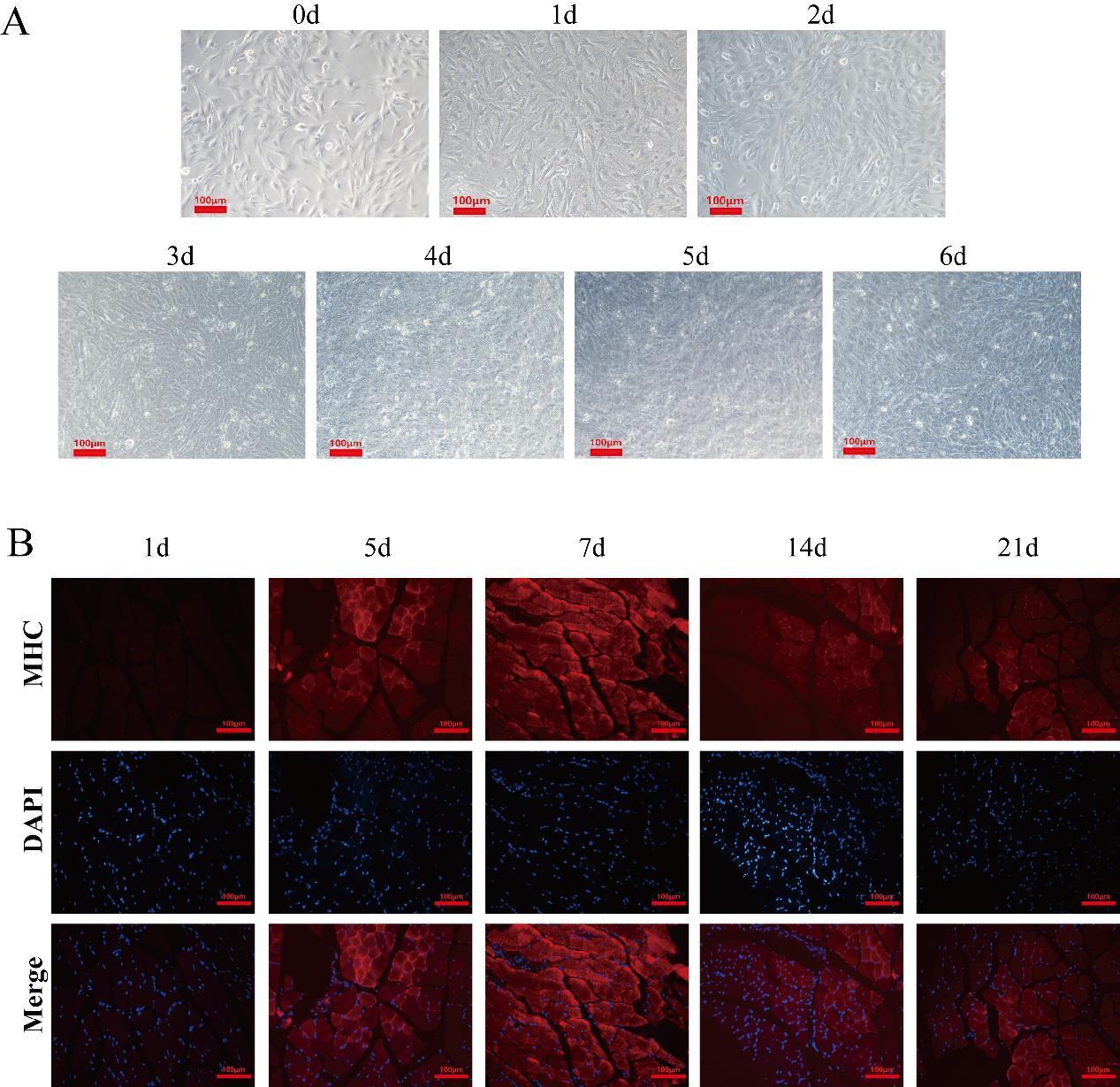
**

**Supplementary Figure 1.** Cell culture and expression of MHC during muscle regeneration of mice.

A: Observing the differentiation of C2C12 cells by a microscope. B: Detection of the expression of MHC (red) in mouse muscle injury by immunofluorescence, and the nuclei were stained with DAPI (blue).


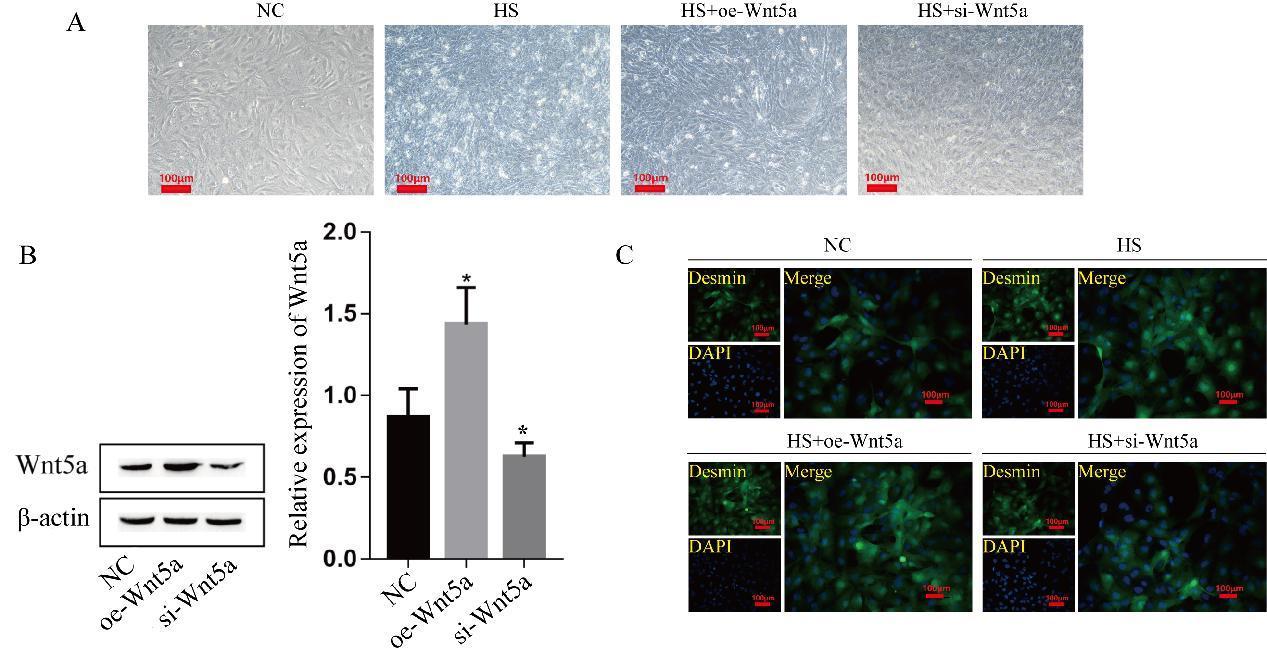


**Supplementary Figure 2.** Role of Wnt5a in myogenic differentiation of C2C12 cells.

A: The status of C2C12 cells after overexpression and knockdown of Wnt5a was observed under the microscope. B: Detection of the transfection efficiency of Wnt5a overexpression and knockdown lentiviral vectors by Western blotting. C: Immunofluorescence was used to detect the expression of Desmin (green), and nuclei were stained with DAPI (blue).


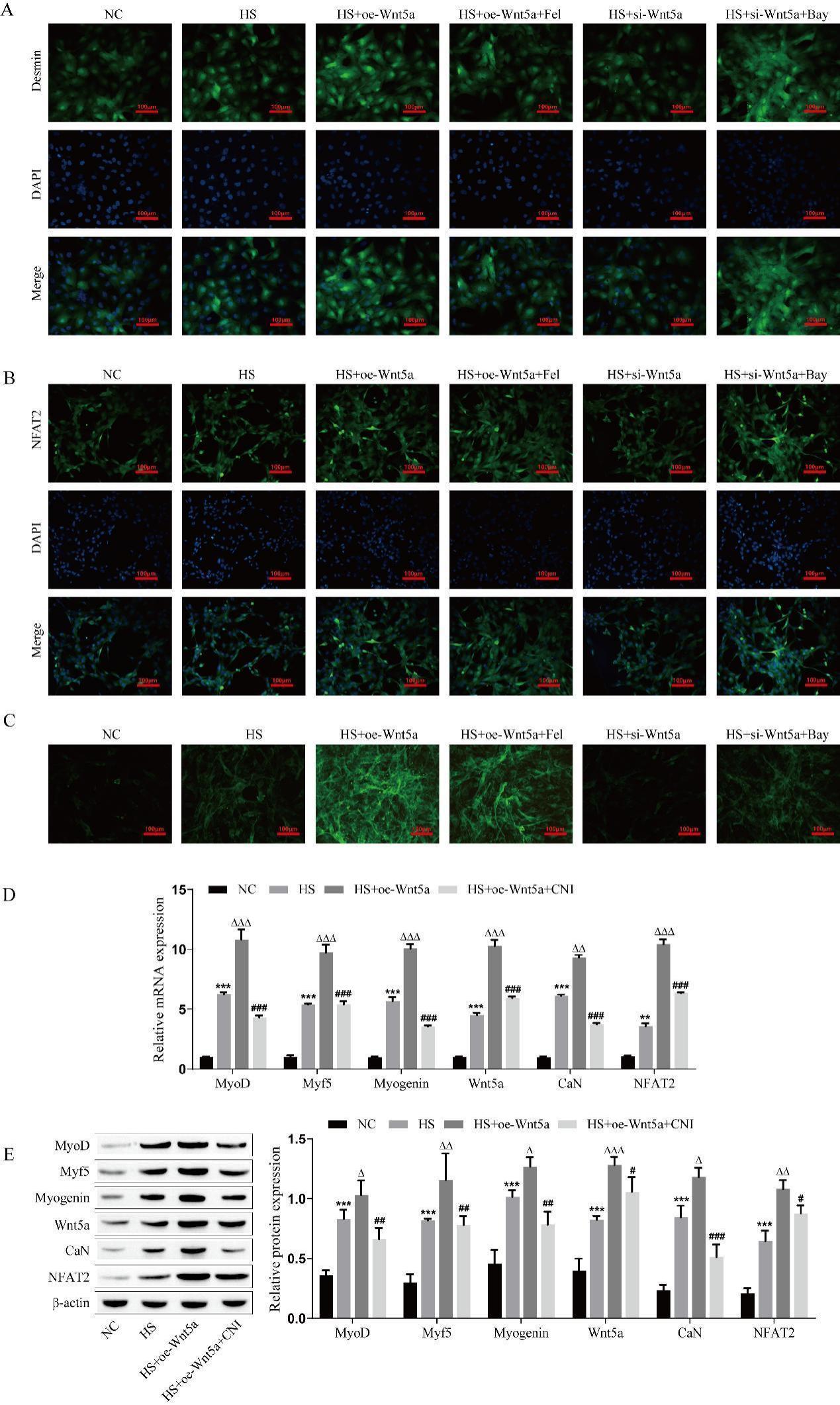


**Supplementary Figure 3.** The role and mechanism of Wnt5a pathway-mediated Ca^2+^ channel opening in C2C12 myoblast differentiation.

A-B: Immunofluorescence for Desmin (green) and NFAT2 (green) expression in C2C12 cells, and nuclei were stained with DAPI (blue). C: The concentration of Ca^2+^ in C2C12 cells was detected by fluorescence microscopy. D: RT-qPCR was performed to detect the mRNA levels of MyoD, Myf5, Myogenin, Wnt5a, CaN, and NFAT2 after the addition of CaN inhibitors (CNI). E: Western blotting was performed to detect the protein levels of MyoD, Myf5, Myogenin, Wnt5a, CaN and NFAT2 after the addition of CaN inhibitors.


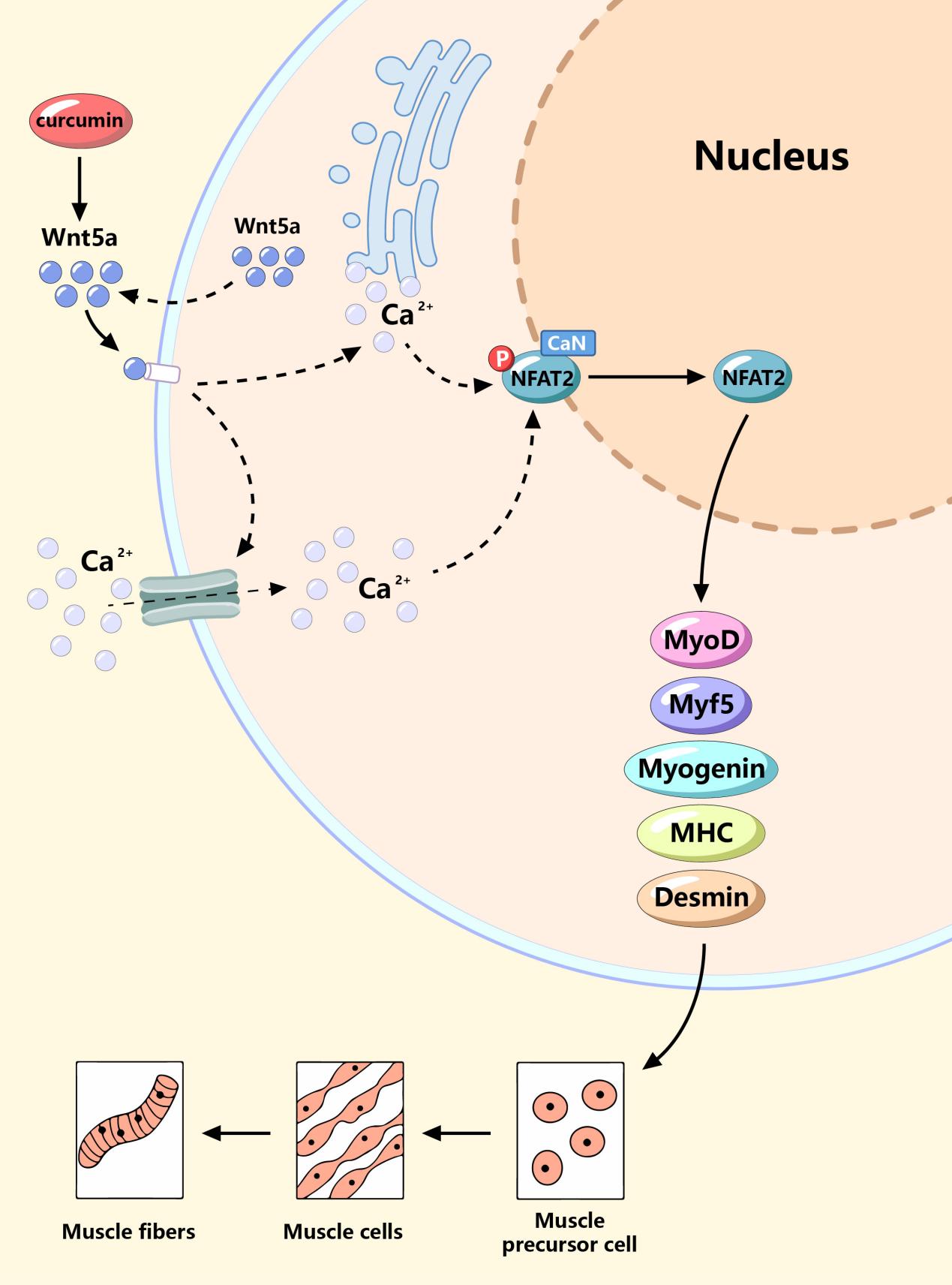


**Supplementary Figure 4.** Schematic diagram of curcumin activation of the Wnt5a signaling pathway to promote myogenic differentiation and muscle regeneration.
